# Fate of Lymphocytes after Withdrawal of Tofacitinib Treatment

**DOI:** 10.1371/journal.pone.0085463

**Published:** 2014-01-09

**Authors:** Elisa Piscianz, Erica Valencic, Eva Cuzzoni, Sara De Iudicibus, Elisa De Lorenzo, Giuliana Decorti, Alberto Tommasini

**Affiliations:** 1 Department of Diagnostic Medicine, Institute for Maternal and Child Health – IRCCS “Burlo Garofolo”, Trieste, Italy; 2 Department of Medical, Surgical and Health Sciences, University of Trieste, Trieste, Italy; 3 Department of Paediatrics, Institute for Maternal and Child Health – IRCCS “Burlo Garofolo”, Trieste, Italy; 4 Department of Translational Research, National Cancer Institute CRO-IRCCS, Aviano, Italy; 5 Department of Life Sciences, University of Trieste, Trieste, Italy; Istituto Superiore di Sanità, Italy

## Abstract

Tofacitinib (Tofa) is an inhibitor of Janus Kinase 3, developed for the treatment of autoimmune diseases and for the prevention of transplant rejection. Due to its selective action on proliferating cells, Tofa can offer a way to block T cell activation, without toxic effects on resting cells. However, few studies have investigated the effects of Tofa on lymphocyte activation *in vitro*. Our aim was to study the action of Tofa on different lymphocyte subsets after *in vitro* stimulation and to track the behaviour of treated cells after interruption of the treatment. Peripheral blood lymphocytes were stimulated *in vitro* with mitogen and treated with two concentrations of Tofa. After a first period in culture, cells were washed and further incubated for an additional time. Lymphocyte subsets, activation phenotype and proliferation were assessed at the different time frames. As expected, Tofa was able to reduce the activation and proliferation of lymphocytes in the first four days of treatment. In addition the drug led to a relative decrease of Natural Killer, B cells and CD8 T cells compared to CD4 T cells. However, treated cells were still viable after the first period in culture and begun to proliferate, strikingly, in a dose dependent manner when the drug was removed from the environment by replacing the culture medium. This novel data does not necessarily predict a similar behaviour *in vivo*, but can warn about the clinical use of this drug when a discontinuation of treatment with Tofa is considered for any reason.

## Introduction

Tofacitinib (Tofa, alternative name CP-690550) is a small molecule inhibitor of Janus Kinase 3 (JAK3), a cytoplasmic tyrosine kinase associated with γc-containing cytokine receptors, involved in signal transduction of interleukin-2 (IL-2), IL-4, IL-7, IL-9, IL-15, IL-21. JAK3 is predominantly expressed in cells of the hematopoietic lineage and plays a key role in the regulation of lymphocyte differentiation, proliferation, survival and apoptosis [1]–[3]. The observation of a severe immunodeficiency in humans and mice with defects in JAK3 highlighted the importance of this signalling pathway in lymphocyte development and activation [4], [5]. An up-regulation of JAK3 has been on the contrary associated with rheumatoid arthritis both in animals and in humans [6]; for these reasons, JAK3 represents a good target for immunotherapy in severe autoimmune disorders.

Thanks to its potency, to its rather specific action and to its good bioavailability after oral administration, Tofa is considered a promising drug for T-cell mediated conditions such as rheumatoid arthritis, inflammatory bowel diseases, psoriasis and organ transplantation. However, available data are still insufficient to predict how Tofa will influence the balance between pathogenic and protective lymphocytes, which is a critical issue for T cell-targeted immunosuppressive therapies. In fact, few data are available on the immunosuppressive action of Tofa *in vitro* and the schedule of *in vivo* administration of the drug is largely empirical.

In this work, we evaluated the behaviour of lymphocytes after *in vitro* treatment with Tofa. We here demonstrated that Tofa strongly blocks T cell activation and proliferation, but, unexpectedly, treated lymphocytes display increased responsiveness to stimulation after the withdrawal of the drug from the culture. Although *in vitro* results do not necessarily predict a similar behaviour *in vivo*, we suggest that caution should be used when discontinuation of treatment with Tofa is considered for any reason. Furthermore, our data could help to develop novel strategies to synchronize and target the activation of pathogenic lymphocytes by exploiting the double-edged immunologic properties of Tofa.

## Methods

### Ethics statement

All the experiments were executed on peripheral blood mononuclear cells (PBMCs) obtained from heparinised blood of healthy donors after written informed consent. The study was approved by the Medical Ethics Review Board of the Institute for Maternal and Child Health – IRCCS “Burlo Garofolo”, Trieste (n.185/08, 19/08/2008) and by the Institutional Research Board (#28/09).

### Drugs, chemicals and reagents

Tofacitinib (Tofa, CP-690550, Selleck Chemical LLC, TX, USA) was dissolved in dimethylsulfoxide (DMSO) and then diluted in the culture medium at a final concentration of 10 (Tofa_10_) or 100 μM (Tofa_100_), so that the final concentration of DMSO would not exceed 1∶320 v/v. These concentrations were chosen on the basis of a previous work [7] and did not induce unspecific cell toxicity, while permitting an effective reduction of proliferation.

Phytohaemagglutinin (PHA, Biochrom AG, Germany) was dissolved in water and then diluted 1∶200 in the culture medium at a final concentration of 1 μg/mL.

Oregon Green 488 carboxylic acid diacetate, succinimidyl ester (Carboxy-DFFDA, SE or CFSE, Invitrogen, Eugene, OR, USA) was dissolved at a concentration of 4.21 mM in DMSO and then used for proliferation assay at a final dye concentration of 5 μM.

7-aminoactinomycin D (7AAD) viability staining was from Immunostep (Salamanca, Spain).

All monoclonal antibodies for flow cytometric analysis were from Miltenyi Biotec (Bergisch Gladbach, Germany) except for CD25 APC-Cy7 (Biolegend, San Diego, CA, USA), HLA DR PE and CD19 FITC (Invitrogen, Carlsbad, CA, USA). 7AAD and monoclonal antibodies were used at concentrations indicated by the manufacturers.

### Experimental plan

On day 0 peripheral blood mononuclear cells (PBMCs) were isolated by Ficoll gradient from heparinised blood of healthy donors and plated at a cell density of 1·10^6^ per millilitre in X-VIVO 15 culture medium (Lonza, Belgium) containing 10% Human Serum AB (Sigma Aldrich, Germany). Cells were stimulated with 1 µg/ml PHA: preliminary experiments using either anti-CD3 and anti-CD28 coated beads or PHA as stimulus, showed that PHA can be a good model to examine Tofa's function on activated T cells. Tofa 10 or 100 µM was simultaneously added to the cell culture. After incubation, on day 4 cells were harvested. PHA and Tofa were washed out and cells were incubated in fresh medium for four additional days (day 4+4). On day 0, day 4 and day 4+4 the distribution of lymphocyte subsets was analysed and analysis of phosphorylation profile of Signal Transducer and Activator of Transcription (STAT) proteins was performed. On day 4 and day 4+4 culture supernatants were collected for cytokine determination and proliferation was assessed at the same time with the expression of the activation marker CD25 and 7AAD viability staining.

### Cell proliferation

Cell proliferation was assessed by means of CFSE dilution after each cell division. CFSE staining was performed either at day 0 or day 4 to evaluate the relative proliferation in the first and second incubation time frame. Briefly, cells were suspended in PBS/BSA 0.5% with 5 µM CFSE, incubated for 10 min at 37°C and then washed three times with cold medium. Cells were then resuspended in complete medium and, after four days in culture, harvested and further stained with the following monoclonal antibodies, in different combination to assess the proliferation of each lymphocyte subpopulation: CD3 PerCP, CD4 APC, CD8 APC-Vio770, CD19 PE-Vio770, CD45 VioBlue, CD56 APC. Cells were also stained with CD25 APC-Cy7 and 7AAD to assess the activation and the viability of proliferated cells.

Data were acquired with Cyan ADP cytometer (Beckman Coulter, Fort Collins, Colorado, USA) and analysis was performed with FlowJo software v 7.6 (TreeStar, Ashland, OR, USA).

### Lymphocyte subsets, activation and viability

At day 0, day 4 and day 4+4, cells were evaluated by flow cytometry to analyze the distribution of lymphocyte subsets. Cells were counted by microscopy using trypan blue viability staining, then 100,000 viable cells were resuspended in saline and stained with different combination of the following antibodies: CD3 FITC, CD4 APC, CD8 APC-Vio770, CD11c FITC, CD16 PE, CD19 PE-Vio770, CD25 APC-Cy7, CD56 PE, CD45 VioBlue, HLA DR PE. Cells were incubated with the antibody mix for 20 min at room temperature, then washed and fixed with FACS Lysing Solution (BD Bioscience, San Jose, CA, USA). T lymphocytes (CD3), B lymphocytes (CD19) and NK cells (CD16/56) were analysed after gating total lymphocytes based on CD45 expression and Side Scatter (SSC) (Figure S1). While this strategy can exclude quite well monocytes and dendritic cells on fresh blood samples, after PHA activation a small quote of monocytes can be included in the analysis. However, only a minor quote of monocytes (less than 3%) can be wrongly included in the natural killer gate, because of the expression of CD16. T helper (CD4) and cytotoxic T cells (CD8) were evaluated after gating on CD3 positive lymphocytes and then CD4/CD8 ratio was calculated. Activated T cells were evaluated by measuring the percentage of CD25 and HLA DR expression after gating on CD45 and CD3 lymphocytes. Activation of B cells was evaluated by measuring the Median Fluorescence Intensity (MFI) of HLA DR after gating on CD45 and CD19 positive cells. Activation of NK cells was evaluated by measuring the percentage of CD11c positive cells after gating on CD45 and CD16/56 cells.

To analyse the viability of each lymphocyte subpopulation, 100,000 cells were stained with CD3 FITC, CD45 VioBlue, CD4 APC, CD8 APC-Vio770 or CD19 FITC, CD45 VioBlue, CD56 APC for 15 min then 7AAD was added and cells were incubated for further 5 min before acquisition by flow cytometry.

The percentage of dead 7AAD positive cells was analysed after gating on CD45 positive lymphocytes and then on each subpopulation.

Data were acquired with Cyan ADP cytometer (Beckman Coulter, Fort Collins, Colorado, USA) and analysis was performed with FlowJo software v 7.6 (TreeStar, Ashland, OR, USA).

### Cytokine profile

Cytokines release was evaluated on culture supernatants samples using a magnetic bead-based multiplex immunoassays (Bio-Plex®) (BIO-RAD Laboratories, Milano, Italy) following manufacturer's instructions. The analysis included 27 cytokines and chemokines: IL-1β, Il-1ra, IL-2, IL-4, IL-5, IL-6, IL-7, IL-8, IL-9, IL-10, IL-12p70, IL-13, IL-15, IL-17A, Eotaxin, basic-FGF, G-CSF, GM-CSF, IP-10, IFN-γ, MCP-1, MIP-1α, MIP-1β, PDGF-BB, RANTES, TNF-α, VEGF. Data were acquired using the Bio-Plex 200 reader, while a digital processor managed data output and Bio-Plex Manager® software presented data as Median Fluorescence Intensity (MFI) and concentration (ng/ml) as well (BIO-RAD Laboratories, Milano, Italy).

### Analysis of phosphoproteins

Phosphorylation of STATs proteins was evaluated using MILLIPLEX MAP-5-Plex STAT panel (Millipore, Billerica, MA, USA), a bead-based multiplex assay. This assay was used to detect changes in phosphorylated STAT1 (Tyr701), STAT2 (Tyr690), STAT3 (Tyr705), STAT5A/B (Tyr694/Tyr699) and STAT6 (Tyr641) in cell lysates obtained from 4·10^6^ PBMC derived cells.

At day 0, day 4 and day 4+4, cells were washed with ice cold PBS containing *cOmplete* protease inhibitors cocktail (Roche Diagnostic, Monza, Italy) and cell pellets were stored at −80°C until analysed. Before analysis, cells were lysed in lysis buffer containing 50 mM sodium fluoride and *cOmplete* protease inhibitor cocktail, then cellular debris were removed by filtration and protein content was measured with RC-DC Protein Assay (Bio-Rad Laboratories, Milano, Italy). The immunoassay was carried out following manufacturer's instructions. Data were acquired using the Lumine*X* 200® reader (Millipore, Billerica, MA, USA), data output managed by *X*-Ponent® software (Millipore, Billerica, MA, USA) and data presented as Median Fluorescence Intensity (MFI).

### Data analysis and statistics

PBMC from a total of eleven healthy donors were analysed in this study, allowing repeating most experiments three or four times.

All results are expressed as mean ± SD. Statistical significance was calculated using one-way analysis of variance (ANOVA) and Bonferroni post-test. Analysis was performed with GraphPad Prism software (version 5.0).

## Results

### Changes in lymphocyte subsets after *in vitro* Tofa treatment

**Figure 1:**
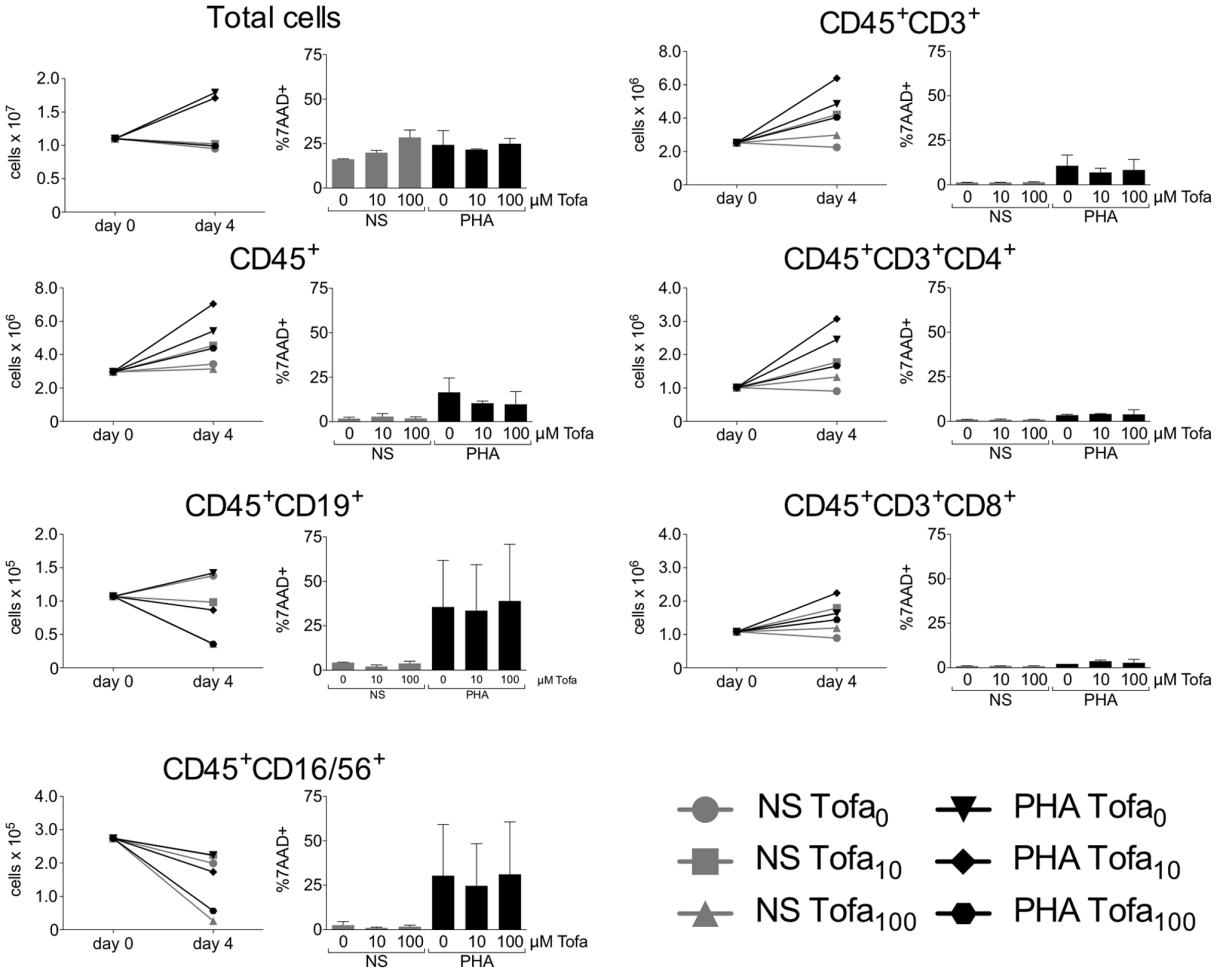
Absolute cell counts and viability. The plots on the left represent the absolute number of cells. Histograms on the right report the percentage of dead cells, as 7AAD positive cells. Counts and viability are reported for each subpopulation of interest, before treatment (day 0) and after the first four days of incubation in presence or absence of PHA and Tofa (day 4). Data are expressed as mean ± SD of two independent experiments.

At day 4, the incubation with Tofa induced a relative decrease in the percentage of B cell (CD19) and NK cell (CD16/56) compared with baseline values (Table 1) both in resting cells and in PHA stimulated cells. This is probably due to an absolute reduction of the cell number because of cell death (Figure 1). In stimulated cells, this effect was even more evident after removal of the drug from culture (day 4+4), but this was likely due to the increase of proliferating CD3.

**Table 1:** Percentages of lymphocyte subsets after Tofa treatment.

		CD3	CD4	CD8	CD4/CD8 ratio	CD19	CD16/56
day 0		70.4±6.2	63.2±8.3	29.0±6.1	2.3±0.7	7.5±3.1	17.1±10.0
day 4	NS Tofa_0_	76.9±8.3	58.4±12.5	31.4±6.5	2.0±0.9	5.7±2.3	12.3±7.6
	NS Tofa_10_	80.6±5.0	60.8±12.2	29.4±5.7	2.2±0.7	5.5±2.3	9.6±4.2
	NS Tofa_100_	87.6±3.8	52.3±23.8	25.7±9.7	2.6±2.0	1.9±0.8	3.6±1.8
	PHA Tofa_0_	85.8±6.6	58.0±10.3	33.0±7.5	1.9±0.6	3.9±2.1	4.6±2.3
	PHA Tofa_10_	83.5±7.4	68.7±9.2	23.6±7.3	3.2±1.1	2.0±1.1	3.1±1.5
	PHA Tofa_100_	84.8±9.1	50.2±21.4	29.9±5.3	1.8±1.0	1.7±0.7	3.0±2.5
day 4+4	NS Tofa_0_	85.5±1.5	61.0±8.6	30.4±4.6	2.1±0.6	5.0±2.3	2.1±1.3
	NS Tofa_10_	86.1±7.9	63.0±9.5	25.7±8.0	2.7±1.0	4.0±2.0	1.2±0.5
	NS Tofa_100_	82.0±11.4	60.5±15.7	21.4±9.9	3.6±2.2	2.3±0.8	0.6±0.4
	PHA Tofa_0_	87.7±1.1	50.1±5.9	43.5±4.0	1.2±0.2	3.5±0.6	5.1±1.7
	PHA Tofa_10_	94.6±1.6	75.5±7.4	22.4±6.9	3.7±1.3	1.3±0.5	0.1±0.0
	PHA Tofa_100_	91.7±4.0	73.5±9.7	22.9±9.7	3.7±1.6	0.9±0.3	0.2±0.1

Cells were analysed before treatment (day 0), after 4 days of incubation with Tofa (day 4) and then four days after the withdrawal of the drug (day 4+4). Percentage of CD3, CD19 and CD16/56 cells were evaluated on total lymphocytes, while CD4 cells, CD8 cells and CD4/CD8 ratio was evaluated on CD3 positive T lymphocytes. Data are expressed as mean ± SD of four independent experiments.

An increased CD4/CD8 T cell ratio was observed in cells stimulated with PHA and treated with Tofa, with higher values reached four days after the removal of the drug from culture. This was probably a result of an increase of CD4 absolute cell number, while CD8 absolute cell number was either unaffected or only slightly increased following Tofa treatment (Figure 1). Besides, a relative decrease of the percentage of CD8 cells rather than to an increase of CD4 cells was observed even in unstimulated (NS) cells treated with Tofa 100 µM (29.0±6.1% at day 0; 25.7±9.7% NS Tofa_100_ at day 4; 21.4±9.9% NS Tofa_100_ at day 4+4) (Table 1).

Both Tofa's concentrations reduced CD19 percentage in PHA stimulated cells, while in NS cells this reduction was evident only with the highest concentration. Moreover, considering cell viability in each lymphocyte subset (Table S1), PHA alone induced a high proportion of dead B and NK cells, which is not different in the presence of Tofa. Due to the small size of these populations, these data need further analyses.

### Inhibition of lymphocyte proliferation during Tofa treatment is reversed with a paradoxical behaviour after removal of the drug

**Figure 2:**
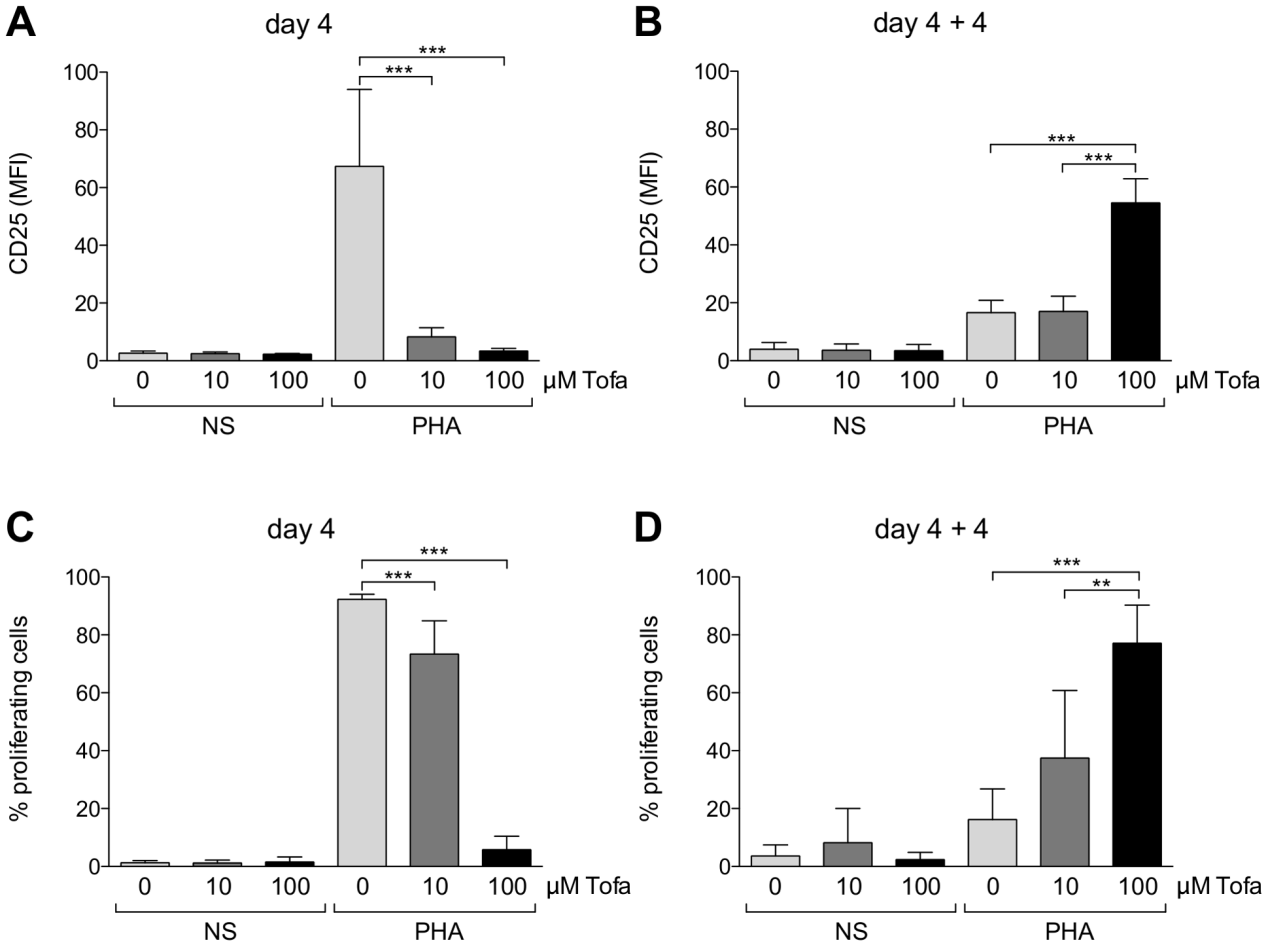
Proliferation and cell activation at the different time frames. Cell activation at day 4 (A) and after four days after withdrawal of Tofa (B) is represented as mean fluorescence index (MFI) of CD25 ± SD of four independent experiments. Cell proliferation at day 4 (C) and after four days after withdrawal of Tofa (D) is represented as percentage of cells with reduced fluorescence of CFSE ± SD of four independent experiments. Statistical significance was calculated using one-way analysis of variance (ANOVA) and Bonferroni post-test. *** p<0.0001, **p<0.001.

As expected, Tofa significantly reduced the proliferation of lymphocytes after PHA stimulation in a dose-dependent manner (92.3±1.7%, 73.4±11.5%, 5.8±4.7% of proliferating cells in Tofa_0_, Tofa_10_ and Tofa_100_ respectively; p<0.0001) (Table 2). The effect of the drug was also confirmed by a significantly reduced expression (p<0.0001) of the activation marker (CD25) on total lymphocytes (Figure 2).

**Table 2:** Lymphocytes activation and proliferation after Tofa treatment.

		% proliferating cells	CD25 MFI
day 4	NS Tofa_0_	1.2±0.8	2.7±0.7
	NS Tofa_10_	1.2±1.0	2.5±0.6
	NS Tofa_100_	1.5±1.8	2.2±0.2
	PHA Tofa_0_	92.3±1.7	67.4±26.7
	PHA Tofa_10_	73.4±11.5	8.2±3.2
	PHA Tofa_100_	5.8±4.7	3.3±0.9
day4+4	NS Tofa_0_	3.6±3.9	3.9±2.3
	NS Tofa_10_	8.2±11.8	3.6±2.1
	NS Tofa_100_	2.4±2.5	3.5±2.2
	PHA Tofa_0_	16.2±10.5	16.6±4.3
	PHA Tofa_10_	37.4±23.3	17.0±5.3
	PHA Tofa_100_	77.1±13.2	54.5±8.4

Cells were analysed after four days of incubation with Tofa (day 4) and four days after the withdrawal of the drug (day 4+4). The percentage of proliferating cells was assessed by measuring the level of CFSE fluorescence. Activation of cells is indicated as MFI of CD25. Data are expressed as mean ± SD of four independent experiments.

In particular, Tofa treatment reduced the expression of activation markers induced by PHA at day 4 only on CD3 lymphocytes with a decreased percentage of CD25 positive (Figure 2, Table 2, Table S2), while the effects were not significant on CD19 (expression of HLA DR) and NK cells (percentage of CD11c positive cells) (Figure 2, Table 2, Table S2).

**Figure 3:**
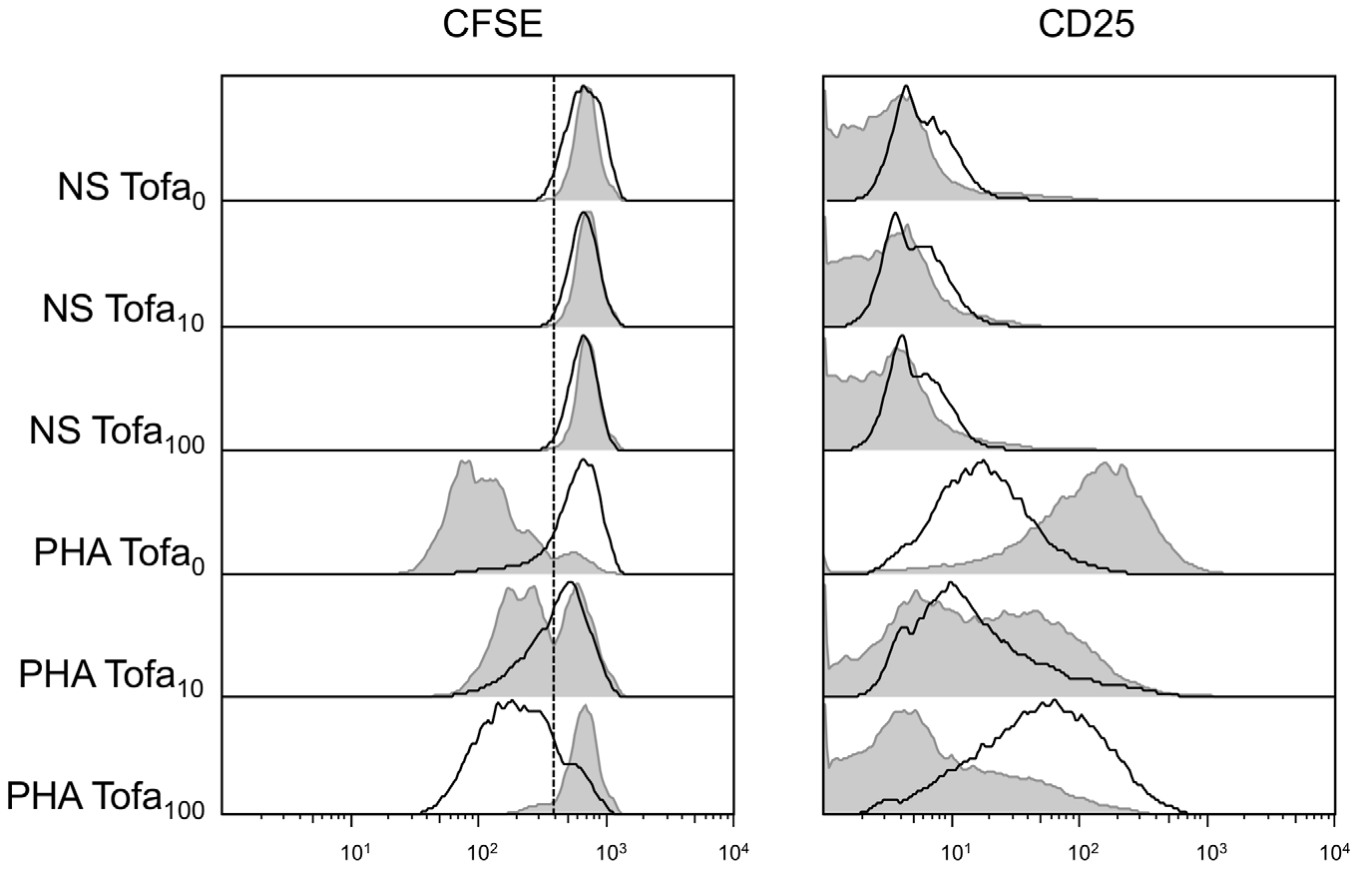
Representative data of cell proliferation and activation. Cells were incubated with PHA and the two concentrations of Tofa (grey histograms); after four days the medium was removed, and the incubation continued in drug-free medium for four additional days (black line histograms). The panel on the left represents the percentage of proliferating cells based on dilution of CFSE dye. On the right expression of CD25 is reported and evaluated as MFI.

However, after removal of the drug from the culture medium (day 4+4), a rescue of lymphocyte proliferation was observed (Figure 2, Table 2), which was surprisingly more consistent in samples that have been previously treated with higher dose of Tofa (16.2±10.5%, 37.4±23.3%, 77.1±13.2% of proliferating cells in Tofa_0_, Tofa_10_ and Tofa_100_ respectively) (a representative experiment is reported in Figure 3) and was paralleled by an increase in activation markers (Figure 2, Table 2, Table S2). In contrast, in cells incubated for the first four days in medium containing PHA but without Tofa, a reduced residual proliferation was observed during the additional four days after medium wash out (4+4). Since cells where CFSE-stained after the first four days of incubation, the proliferation assay evaluated only the proliferation in the second time frame of culture. A subset of experiments, performed on cells stained at time 0, showed that cells treated with Tofa_10_ displayed a proliferation at day 4+4 similar to cells not treated with the drug, whilst a slight overall decrease of proliferation was achieved by cells treated with Tofa_100_ (data not shown). These results indicate that all the cells blocked during the first four days of Tofa administration are ready to proliferate when the drug is washed out.

### Inhibition of cytokine release

**Figure 4:**
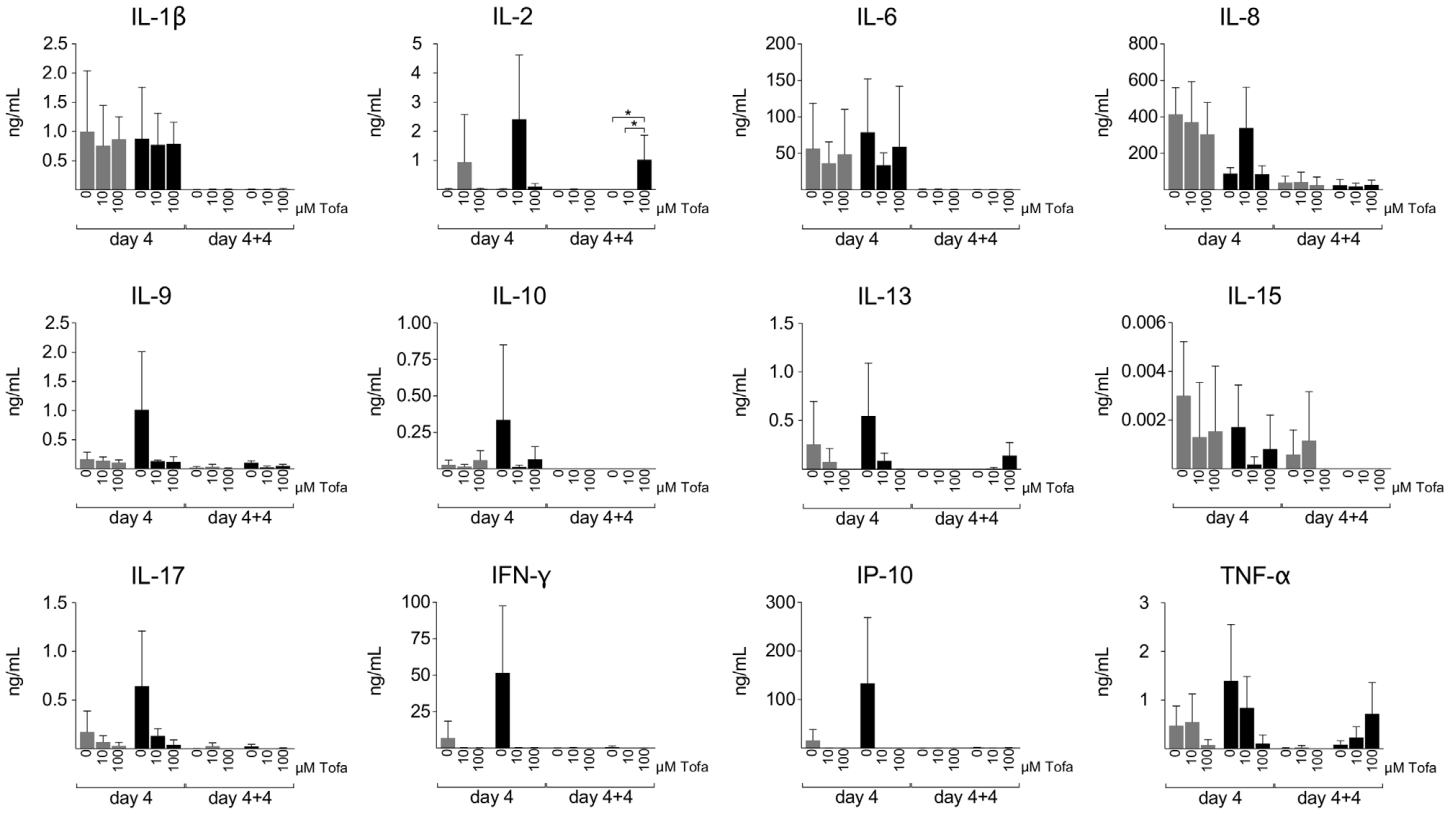
Cytokine determination in culture supernatants at different time frames. Cytokines were evaluated by magnetic bead-based multiplex immunoassays. The analysis was performed at day 4 and at day 4+4 on three series of culture supernatants. Data are expressed as mean concentration (ng/mL) ± SD. Only the histograms of the cytokines mainly involved in the effects evaluated in the study are reported in the figure. Grey bars: unstimulated cells; Black bars: PHA stimulated cells. Statistical significance was calculated using one-way analysis of variance (ANOVA) and Bonferroni post-test. *p<0.05.

During the first four days of culture, Tofa treatment inhibited the secretion of IL-2, IL-9, IL-10, IL-13, IL-17, TNF-α in a dose-dependent manner. In contrast, at day 4+4, Tofa-treated cells showed an increased release only of IL-2 (p<0.05), TNF-α and IL-13 that was particularly evident in cells treated with Tofa_100_. Besides, a complete depletion of IFN-γ and IP-10 was seen despite the proliferation evidenced in PHA+Tofa_100_ treated cells (Figure 4). The reduced concentration of IFN-γ and its related product (IP-10) could be a result of the loss of the NK population.

Other cytokines, mainly secreted by monocytes, such as IL-1β, IL-6 and IL-8 were less influenced by the treatment in the first 4 days and decreased regardless of the treatment in the longer incubation time (day 4+4) (Figure 4).

### Phosphorylation profile of STAT proteins

After the first four days of culture, STAT1 and STAT2 phosphorylation showed an activation-induced increase that was inhibited in a dose dependent manner by Tofa (significant for STAT1 p<0.05). Conversely, STAT3 phosphorylation seemed to be inhibited by Tofa both in NS and in PHA stimulated cells. Only a slight trend toward a lower STAT5 phosphorylation was observed in PHA stimulated cells after Tofa treatment, while STAT6 phosphorylation didn't show appreciable changes.

**Figure 5:**
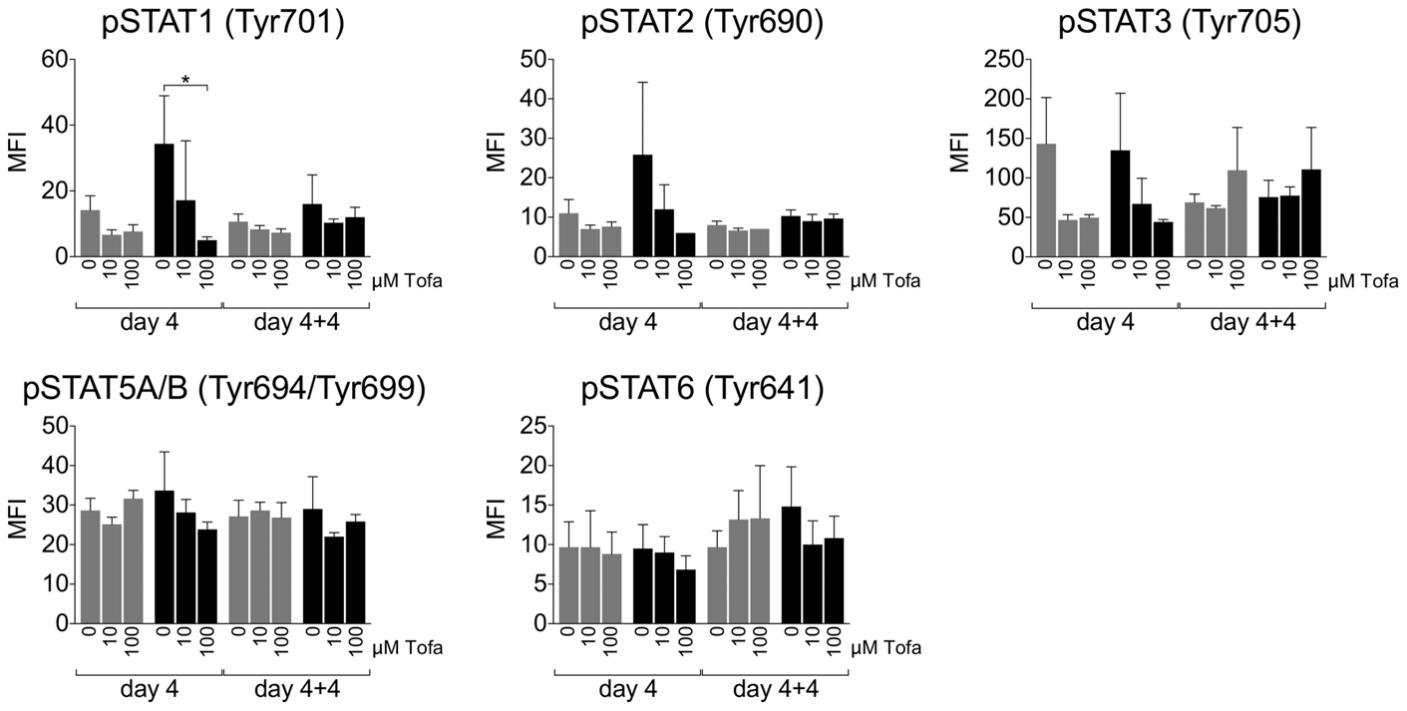
Phosphorylation profile of STAT proteins. Phosphorylation profile of STAT1, STAT2, STAT3, STAT5A/B, STAT6 was evaluated by magnetic bead-based multiplex immunoassays. Data are expressed as Median Intensity Fluorescence (MFI) ± SD of three independent experiments. Grey bars: unstimulated cells; Black bars: PHA stimulated cells. Statistical significance was calculated using one-way analysis of variance (ANOVA) and Bonferroni post-test. *p<0.05.

During the second period of incubation (day 4+4) the phosphorylation profiles of all the tested STAT proteins remained stable without any appreciable change regardless of the treatment of the cells (Figure 5).

## Discussion

Tofacitinib is a potent immunomodulatory drug belonging to the category of small molecules targeting kinase signalling [8]. Based on its mechanism of action, Tofa can fit into the tool box of drugs acting on lymphocyte activation, which already includes calcineurin inhibitors, mammalian target of rapamycin (mTOR) inhibitors and co-stimulation targeting biologics [9]. The potential field of clinical application is thus represented by disorders with inappropriate T cell response, such as rheumatoid arthritis, psoriasis, graft rejection, ulcerative colitis and graft versus host disease.

Compared to other drugs acting on lymphocyte activation, Tofa was intended to offer higher efficacy, due to its action on different lymphotropic cytokines, with a safer profile, being the expression of target kinases restricted to lymphoid cells [10].

However, the tuning of efficacy (suppression of pathogenic lymphocytes) and safety (suppression of protective immunity) remains an open issue. In fact, serious and sometimes fatal infections and immunosuppression-related malignancies have been reported in patients with rheumatoid arthritis receiving Tofa [11], [12]. Furthermore, the action of the drug in long term therapies, alone or in combination with other drugs, and the evolution of diseases after drug withdrawal, have not been thoroughly explored. Unexpectedly, whereas the drug has been widely used in animal models and has been already introduced into the clinics, only few studies had investigated its immunological potential *in vitro*.

Here we showed that Tofa exerts a rapid and strong effect on PHA stimulated lymphocytes, leading to a complete arrest in proliferation and to a strong down-regulation of activation markers. Notably, these results are achieved with a negligible toxicity on T lymphocyte viability assessed by 7AAD. However, the drug induces a redistribution of lymphocyte subsets by increasing the death of B cells and NK cells and by a stronger inhibition of CD8 compared to CD4 T cells. In other studies, anti-T cell receptor antibody-induced proliferation of *naïve* CD4 cells was not affected by the addition of Tofa at doses between 10 and 100 ng/ml, which are in the range of the pre-dose levels of the drug in peripheral blood of treated patients [18], [20], [21]. In contrast, we showed that a near complete block in proliferation can be obtained by using much higher doses of Tofa (100 µM), still without appreciable cytotoxicity.

What is completely new, is that, after the withdrawal of the drug, stimulated T lymphocytes resumed proliferation. Thus, transient treatment with Tofa doesn't lead to a relevant inhibition of final proliferation, but it still strongly affects the distribution of lymphocyte subsets, with a reduction of NK cells, B cells and CD8 cells.

Although *in vitro* PHA stimulus induces direct activation only on T cells, the bystander effects due to this activation clearly shows its side-effect also on other cell compartments. Besides, Tofa treatment could even impact the distribution of cell populations and this is coherent with the phenotype commonly found in JAK3-Severe Combined Immunedeficiency (SCID) in which a decrease of NK and CD8 is observed [13], [14]. More unexpected was the loss of B cells that is not a typical finding *in vivo*, both in T^−^B^+^NK^−^ JAK3-SCID and in subjects treated with Tofa. Indeed, as reported by others [13]–[15] the survival of B cells *in vivo* may be ascribed to different mechanisms, such as an increased differentiation of mature B cells in the bone marrow, a compensatory IL-3-based mechanism [15]. Furthermore, B cells are dependent upon CD40L stimulation that, *in vivo*, can be provided not only by activated T cells but also by dendritic cells [16].

To the best of our knowledge, the finding that replenishment of Tofa from culture resulted in the rebound of suppressed T cell proliferation has not been previously described. Of course, these data may reflect just a particular experimental setting *in vitro*, but they may be relevant to answer unsolved questions about the immunomodulatory action of Tofa and not-infectious adverse events observed in the clinics [17]. Indeed, the lower dosage used in our study is close to the peak concentration occurring in patients' serum during treatment (Tofacitinib for Treatment of Rheumatoid Arthritis (NDA 203214) Advisory Committee Meeting).

Other authors evaluated the response to cytokine stimulation in the presence of Tofa, showing a variable impact on JAK signalling, as confirmed by changes in STAT proteins phosphorylation, accounting for a preferential action on Th1 and Th17 lineages [18], [19]. On the contrary, low dose Tofa led to the worsening of experimental encephalomyelitis *in vivo* and resulted in increased Th17 differentiation *in vitro* [20].

To evaluate the impact of Tofa and its replenishment on JAK signaling, we studied the production of different cytokines and the phosphorylation of different STAT proteins downstream JAK kinases.

Of note, while a Tofa-dependent reduction of activation-induced phosphorylation was evident for STAT1 and STAT3, a lower effect could be evidenced on STAT5, which is the major target of JAK3 signalling, confirming a broader effect of the drug on different JAK-STAT pathways. In contrast, the only STAT protein that showed a trend increase in phosphorylation after the second period of incubation was STAT3, suggesting a possible role of JAK1-STAT3 pathway in the proliferation rebound observed after withdrawal of the drug. However, STAT3-dependent cytokines such as IL-17 remained suppressed by the treatment with Tofa also after the second incubation time frame and lymphocytes rescued to proliferate only produced IL-2, IL-13 and TNF-α, raising the possibility of a modulation of their Th1/Th2 profile.

In other studies, responsiveness of lymphocytes to cytokines stimulation was shown to be restored after Tofa withdrawal, however the drug was used at lower dosages and the residual lymphocyte proliferation was not investigated [18].

Based on these data, we can presume that discontinuation of the drug after a short treatment may lead to reactivation of diseased lymphocytes also *in vivo*, possibly resulting in undesired effects of the drug. In addition, a reduction of NK cells has been already reported in clinical trials [11] and may contribute to the increased risk of infections and malignancies. To evaluate this possibility, a careful study of the expression of lymphocyte activation markers and of the distribution of lymphocyte subsets should be performed in all subjects after discontinuation of Tofa treatment. Moreover, we think that further studies are needed to evaluate the sensitivity of Tofa-treated lymphocytes to other immunosuppressive drugs: this would be useful to allow a safe discontinuation of the drug and to develop optimized schedules for using Tofa in combination with other drugs.

## Supporting Information

Figure S1 **Gating strategy to identify lymphocyte subpopulations.** Lymphocytes were identified based on the side scatter and high CD45 expression (A); T lymphocytes were identified as CD3 positive cells (B and C, lower right); NK cells were identified as CD16/56 positive cells (B, upper left); B cells were identified as CD19 positive cells (C, upper left). Among CD3 T cells, CD4 positive (D, lower right) cells were separated from CD8 positive cells (D, upper left).
(TIFF)

Table S1 **Percentage of dead cells in each lymphocyte subset.** Dead cells was evaluated as percentage of 7AAD positive cells, before treatment (day 0), after stimulation with PHA and treatment with Tofa (day 4) and after withdrawal of the drug (day 4+4). Data represent the mean ± SD of two independent experiments.
(DOCX)

Table S2 **Evaluation of activation markers on the different lymphocyte subsets.** Activation markers were evaluated at each time frames. Activation of CD3 cells was evaluated as percentage of CD25 and HLA DR positivity; activation of CD19 cells was evaluated as variation in Median Fluorescence Intensity (MFI); activation of NK cells was evaluated as percentage of CD11c positivity. Data report the mean ± SD of two independent experiments.
(DOCX)
