# Supplementary figures and images for: Fate of Lymphocytes after Withdrawal of Tofacitinib Treatment
Source: PLoS One. 2014 Jan 9;9(1):e85463. doi: 10.1371/journal.pone.0085463 (PMC3887061; doi:10.1371/journal.pone.0085463)

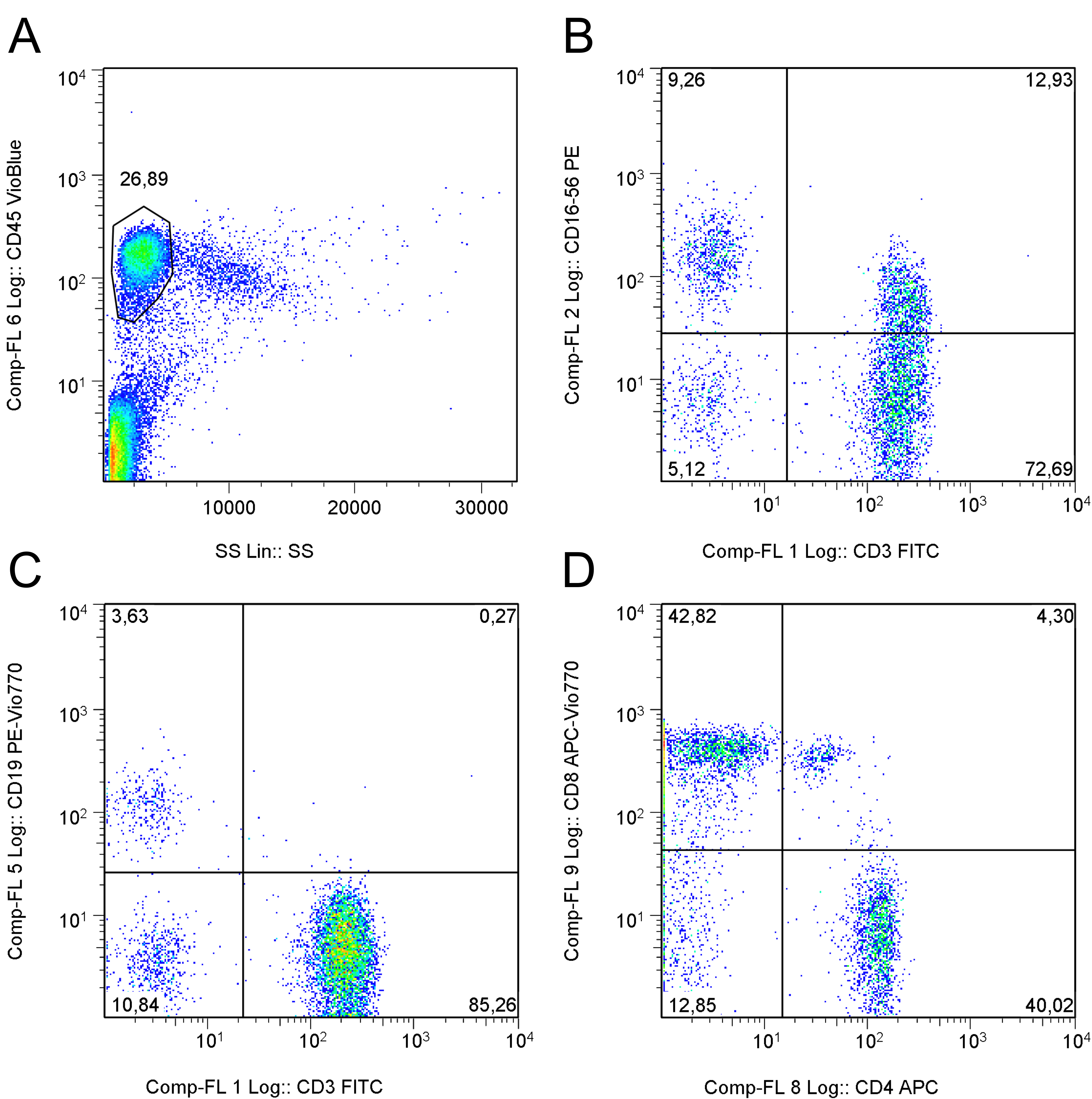

Supplement: Figure S1 — Gating strategy to identify lymphocyte subpopulations. Lymphocytes were identified based on the side scatter and high CD45 expression (A); T lymphocytes were identified as CD3 positive cells (B and C, lower right); NK cells were identified as CD16/56 positive cells (B, upper left); B cells were identified as CD19 positive cells (C, upper left). Among CD3 T cells, CD4 positive (D, lower right) cells were separated from CD8 positive cells (D, upper left). (TIFF) [file pone.0085463.s001.tif]
