# Supplementary material for: Fate of Lymphocytes after Withdrawal of Tofacitinib Treatment
Source: PLoS One. 2014 Jan 9;9(1):e85463. doi: 10.1371/journal.pone.0085463 (PMC3887061; doi:10.1371/journal.pone.0085463)
Supplement: Table S1 — Percentage of dead cells in each lymphocyte subset. Dead cells was evaluated as percentage of 7AAD positive cells, before treatment (day 0), after stimulation with PHA and treatment with Tofa (day 4) and after withdrawal of the drug (day 4+4). Data represent the mean ± SD of two independent experiments. (DOCX) [file pone.0085463.s002.docx]

**Table S1. Percentage of dead cells in each lymphocyte subset.**

|  |  | **CD3** | **CD4** | **CD8** | **CD19** | **NK** |
| --- | --- | --- | --- | --- | --- | --- |
| day 0 |  | 1.44 ± 0.25 | 1.77 ± 0.08 | 1.33 ± 0.13 | 10.46 ± 1.28 | 3.61 ± 1.11 |
| day 4 | NS Tofa_0_ | 1.25 ± 0.12 | 1.03 ± 0.15 | 1.03 ± 0.15 | 4.35 ± 0.19 | 2.62 ± 1.96 |
|  | NS Tofa_10_ | 1.36 ± 0.04 | 0.99 ± 0.28 | 1.10 ± 0.06 | 2.15 ± 0.89 | 1.08 ± 0.37 |
|  | NS Tofa_100_ | 1.48 ± 0.24 | 0.90 ± 0.17 | 0.86 ± 0.25 | 3.87 ± 1.24 | 1.78 ± 0.74 |
|  | PHA Tofa_0_ | 10.74 ± 5.95 | 3.49 ± 0.48 | 2.15 ± 0.02 | 35.59 ± 26.28 | 30.38 ± 28.80 |
|  | PHA Tofa_10_ | 6.94 ± 2.39 | 4.18 ± 0.22 | 3.67 ± 0.62 | 33.54 ± 25.85 | 24.70 ± 23.62 |
|  | PHA Tofa_100_ | 8.34 ± 6.00 | 3.89 ± 2.61 | 2.87 ± 1.82 | 38.94 ± 31.92 | 31.18 ± 29.48 |
| day4+4 | NS Tofa_0_ | 1.57 ± 0.96 | 1.23 ± 0.99 | 1.03 ± 0.54 | 3.40 ± 2.01 | 1.71 ± 0.43 |
|  | NS Tofa_10_ | 1.19 ± 0.90 | 0.97 ± 0.72 | 0.74 ± 0.57 | 3.39 ± 1.67 | 1.64 ± 0.31 |
|  | NS Tofa_100_ | 1.21 ± 0.84 | 1.31 ± 1.02 | 0.91 ± 0.72 | 20.82 ± 10.93 | 4.30 ± 2.27 |
|  | PHA Tofa_0_ | 2.23 ± 0.56 | 2.00 ± 0.71 | 1.65 ± 1.29 | 20.77 ± 7.80 | 10.74 ± 8.62 |
|  | PHA Tofa_10_ | 3.13 ± 0.93 | 2.86 ± 0.65 | 2.43 ± 0.14 | 14.94 ± 7.88 | 8.56 ± 6.42 |
|  | PHA Tofa_100_ | 2.76 ± 0.70 | 3.09 ± 0.14 | 1.72 ± 0.52 | 28.20 ± 20.24 | 15.98 ± 14.00 |

Dead cells was evaluated as percentage of 7AAD positive cells, before treatment (day 0), after stimulation with PHA and treatment with Tofa (day 4) and after withdrawal of the drug (day 4+4). Data represent the mean ± SD of two independent experiments.
