# Supplementary material for: Fate of Lymphocytes after Withdrawal of Tofacitinib Treatment
Source: PLoS One. 2014 Jan 9;9(1):e85463. doi: 10.1371/journal.pone.0085463 (PMC3887061; doi:10.1371/journal.pone.0085463)
Supplement: Table S2 — Evaluation of activation markers on the different lymphocyte subsets. Activation markers were evaluated at each time frames. Activation of CD3 cells was evaluated as percentage of CD25 and HLA DR positivity; activation of CD19 cells was evaluated as variation in Median Fluorescence Intensity (MFI); activation of NK cells was evaluated as percentage of CD11c positivity. Data report the mean ± SD of two independent experiments. (DOCX) [file pone.0085463.s003.docx]

**Table S2. Evaluation of activation markers on the different lymphocyte subsets.**

|  |  | **CD3 cells** | **CD3 cells** | **CD19 cells** | **NK cells** |
| --- | --- | --- | --- | --- | --- |
|  |  | **% CD25^+^** | **% HLA DR^+^** | **MFI HLA DR^+^** | **% CD11c^+^** |
| day 0 |  | 6.98 ± 0.85 | 2.02 ± 0.66 | 118.34 ± 20.94 | 9.90 ± 5.14 |
| day 4 | NS Tofa_0_ | 3.42 ± 0.08 | 1.13 ± 0.12 | 213.76 ± 79.34 | 13.94 ± 7.08 |
|  | NS Tofa_10_ | 1.82 ± 0.54 | 2.32 ± 1.32 | 247.91 ± 91.82 | 28.33 ± 2.84 |
|  | NS Tofa_100_ | 1.51 ± 0.15 | 2.16 ± 0.69 | 362.84 ± 207. 24 | 20.30 ± 16.57 |
|  | PHA Tofa_0_ | 52.67 ± 16.59 | 8.31 ± 2.13 | 299 ± 25.52 | 62.68 ± 26.22 |
|  | PHA Tofa_10_ | 10.52 ± 1.86 | 7.29 ± 2.77 | 519.05 ± 22.33 | 66.18 ± 28.33 |
|  | PHA Tofa_100_ | 4.60 ± 0.62 | 5.22 ± 1.61 | 517.80 ± 216.51 | 47.93 ± 41.70 |
| day4+4 | NS Tofa_0_ | 5.27 ± 0.19 | 3.80 ± 2.72 | 165.38 ± 48.87 | 45.89 ± 26.42 |
|  | NS Tofa_10_ | 2.07 ± 0.81 | 0.76 ± 0.14 | 181.13 ± 46.08 | 60.27 ± 29.18 |
|  | NS Tofa_100_ | 1.72 ± 0.41 | 1.63 ± 0.34 | 278.77 ± 84.32 | 50.18 ± 42.39 |
|  | PHA Tofa_0_ | 33.58 ± 11.92 | 8.12 ± 2.06 | 316.51 ± 9.32 | 69.57 ± 20.67 |
|  | PHA Tofa_10_ | 50.44 ± 31.65 | 24.37 ± 14.95 | 325.88 ± 4.29 | 81.19 ± 10.94 |
|  | PHA Tofa_100_ | 25.52 ± 22.24 | 5.81 ± 0.63 | 387.15 ± 74.60 | 71.82 ± 18.38 |

Activation markers were evaluated at each time frames. Activation of CD3 cells was evaluated as percentage of CD25 and HLA DR positivity; activation of CD19 cells was evaluated as variation in Median Fluorescence Intensity (MFI); activation of NK cells was evaluated as percentage of CD11c positivity. Data report the mean ± SD of two independent experiments.
